# Sigmoid volvulus in pregnancy: a case report

**DOI:** 10.1186/s13256-021-03151-3

**Published:** 2021-11-10

**Authors:** Jay Lodhia, Joachim Magoma, Joylene Tendai, David Msuya, Jamil Suleiman, Kondo Chilonga

**Affiliations:** 1 Department of General Surgery, Kilimanjaro Christian Medical Centre, P O Box 3010, Moshi, Tanzania; 2 Kilimanja Christian Medical University College, P O Box 2240, Moshi, Tanzania

**Keywords:** Intestinal obstruction, Pregnancy, Sigmoid volvulus, Tanzania

## Abstract

**Introduction:**

Sigmoid volvulus in pregnancy is a rare cause of intestinal obstruction with high maternal and fetal morbidity and mortality if not diagnosed and managed early.

**Case presentation:**

A 29-year-old female (Chagga by tribe) presented with clinical features of intestinal obstruction 24 weeks into her second pregnancy. She had symptoms for one week. An emergency laparotomy was performed whereby gangrenous sigmoid volvulus was found; thus, it was resected and Hartmann’s colostomy was raised. Unfortunately, she experienced intrauterine fetal death post-operatively. She was discharged clinically stable.

**Conclusion:**

Early diagnosis and management can prevent adverse effects such as bowel ischemia and preterm labor. Because classic clinical and radiological features may not be evident, high degree of suspicion is warranted.

## Background

Sigmoid volvulus is a rare cause of intestinal obstruction in pregnancy, with incidence ranging from 1 in 1500 to 1 in 66,431 deliveries [1]. Other common causes include adhesions, hernia, malignancies, and intussusceptions [2]. This pathology is associated with high maternal and fetal complications due to the delayed presentations, and accurate diagnosis is difficult to make because of the normal anatomical and physiological changes during pregnancy [2]. Overall outcome depends on timely diagnosis and management. We present a young female who, in her second trimester, presented with intestinal obstruction (IO) that was found to be caused by sigmoid volvulus intraoperatively.

## Case presentation

A 29-year-old female (Chagga by tribe) 24 weeks pregnant was referred from the regional hospital to our center (a referral hospital) with a 1-week history of abdominal pain and constipation. The pain started gradually and was generalized and cramping in nature. It was associated with vomiting containing food material. She denied history of fever. She reported a decrease in fetal movements and denied any history of abdominal trauma or vaginal discharge. Her past medical history was insignificant. This was her second pregnancy; the first was delivered by cesarean section because of preeclampsia, and the child is growing well. For her index pregnancy, she was on iron and folate supplements.

Upon examination, she was ill looking, conscious, alert, mildly pale, and dehydrated, with a temperature of 38 ℃ and a nasogastric tube (NGT) *in situ* draining fecal content. Her blood pressure (BP) was 112/81 mmHg, pulse 136 beats per minute, and saturation 96% on room air. Her abdomen was symmetrically distended and moving with respiration, had a Pfannenstiel incision scar, and was tense and tender on palpation with a symphysiofundal height correlating to 23 weeks. Muscle guarding could not be elicited because of the tense abdomen, and hypertympanic note on percussion and no bowel sounds were heard on auscultation. There was nothing abnormal detected on rectal and vaginal examination. Other systems were unremarkable.

**Fig. 1:**
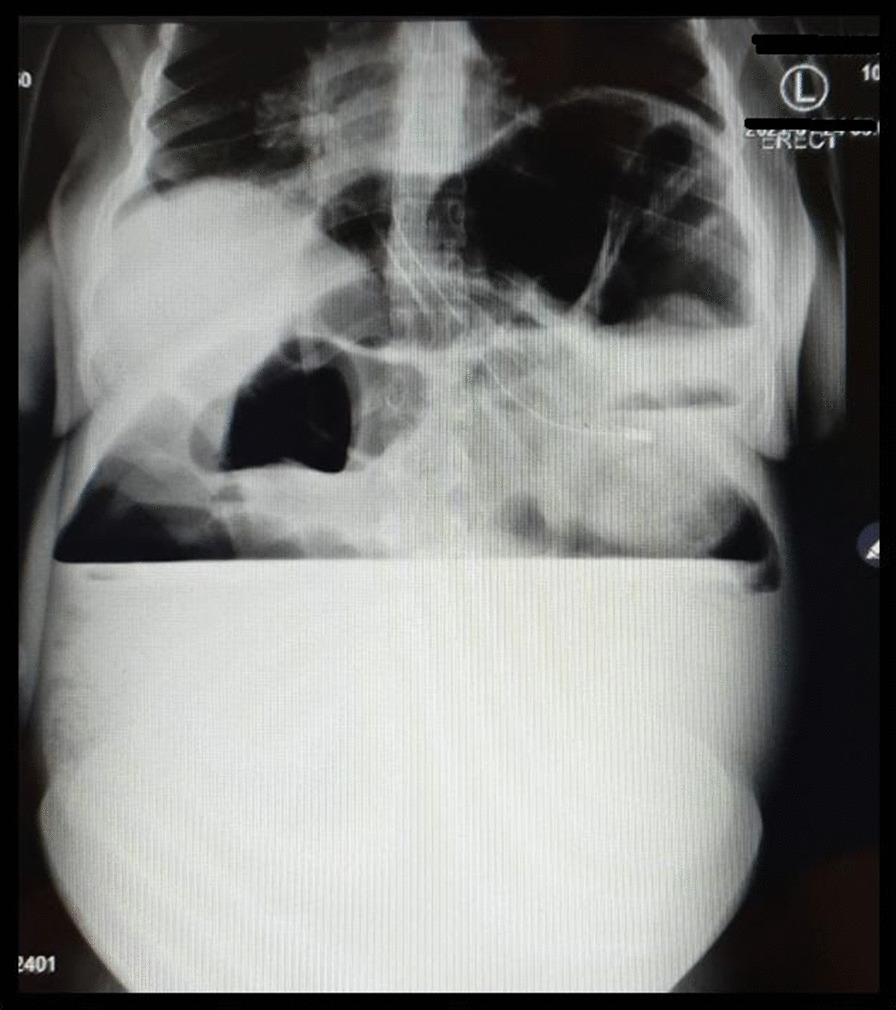
Erect abdominal X-ray showing high air–fluid level and distended gastric bubble

Her complete blood count on admission showed a normal leukocyte count of 9.17 × 10^9^/L, anemia of 9.4 g/dl, and platelet count of 441 × 10^9^/L. She had mild hypokalemia of 2.52 mmol/L, which was corrected by intravenous potassium chloride. Initial abdominal ultrasonography revealed gaseous abdomen with a viable intrauterine pregnancy of 24 weeks. Abdominal X-ray was done that was suggestive of intestinal obstruction with a differential of perforated hollow viscus (Fig. 1). She was kept nil orally and on intravenous fluids for resuscitation.

**Fig. 2:**
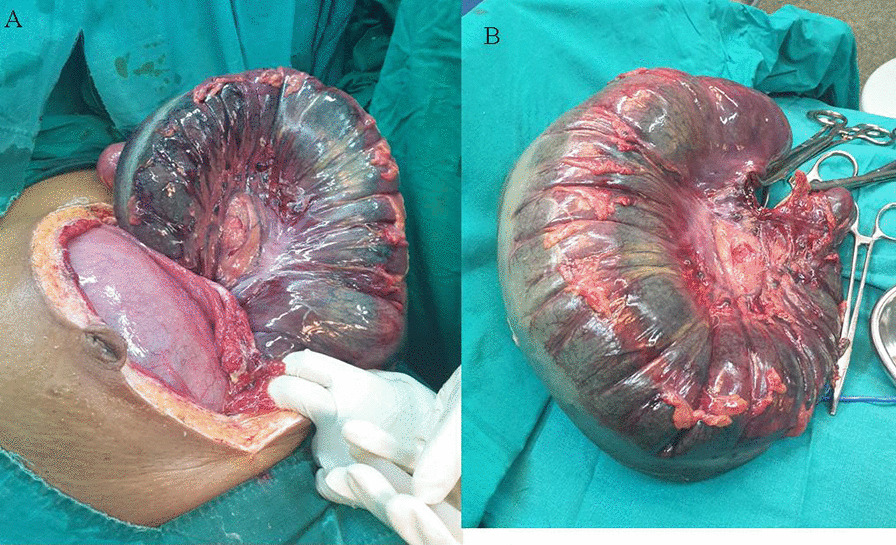
**A** Gangrenous sigmoid volvulus and gravis uterus. **B** Grossly dilated sigmoid colon post resection

She was consented for an emergency laparotomy whereby the abdomen was opened through a midline incision. A gravid uterus, approximately 500 ml of amber-colored ascites, and a 360° anticlockwise sigmoid volvulus around its mesentery, which was gangrenous and distended, were found (Fig. 2). The bowels proximal to the volvulus were distended. Thus, derotation of the volvulus and resection of sigmoid colon were done followed by a Hartman’s colostomy. Hemostasis was achieved, abdominal lavage was done, and the abdomen was closed in layers. She received 450 milliliters of whole blood intraoperatively. She was then nursed at the intensive care unit, where on day one she expelled a male fetus weighing 800 g with no signs of life.

During her stay in ICU, she was kept on intravenous antibiotics, and was transfused with one more unit of blood. Her control hemoglobin was 11.5 g/dl. On day 7 postoperatively, she continued to do well clinically with a functioning colostomy and was thus discharged home with instructions on colostomy care and to continue follow-up as an outpatient. Six weeks post discharge, she was reviewed at the outpatient clinic, where she was clinically stable with functioning colostomy and unremarkable histology for the sigmoid colon; she was scheduled for an elective colostomy closure after a normal distal loopogram (barium enema). She was also reviewed by the obstetrics and gynecology team and started her on oral combined contraceptive pills to regulate her menstrual cycle. Six weeks post-colostomy closure, she was clinically stable with no abdominal complaints and, hence, discharged.

## Discussion

Sigmoid volvulus is a rare cause of intestinal obstruction in pregnancy with high maternal and fetal mortality [2]. It is said to be caused by a redundant sigmoid colon, high-fiber diet (attributed to African origin), chronic constipation, and pregnancy, especially in the third trimester, owing to the displacement and partial compression of the sigmoid colon by the gravid uterus [2, 3]. In our case, it appears that the sigmoid was displaced and compressed by the gravid uterus, causing sigmoid volvulus, though redundant sigmoid cannot be ruled out.

Classic presentation of sigmoid volvulus is abdominal distension, constipation, and abdominal pain, which were present in the index case; however, obstructive symptoms can be unspecific in pregnancy as they can be related to the pregnancy. Nevertheless, common features of IO in pregnancy include abdominal pain (98%), vomiting (82%), and constipation (30%) [1, 2, 4]. Vomiting was a strong feature in our case as the NGT was draining fecal contents to support the diagnosis of IO. Generally, these women are 15–35 years of age and 75% are multiparous and 66% in their third trimester, all of which was true for our case [1]. Other causes of IO in pregnancy include appendicitis, intraabdominal neoplasms, adhesions from previous surgeries, intussusceptions, and hernias [3].

Delay in the diagnosis hence leads to delay in the management, culminating in devastating outcomes; 5% maternal mortality has been reported if the bowels are viable and over 50% if perforation occurs. Fetal mortality is said to be 30% [2]. Other maternal complications include perforation, peritonitis, and sepsis, and fetal complications include preterm delivery, intrauterine fetal death, and neonatal sepsis [4].

Clinical examination is limited due to the gravid uterus, and radiological evaluation presents another challenge due to the risks of teratogenicity of the fetus. In our case, the age of pregnancy was 24 weeks, and thus reduced risk, whereas generally the health of the mother takes priority over the fetus [2]. Plain abdominal X-ray has a sensitivity of 90% in diagnosing IO, whereas ultrasound can show dilated bowels and maybe show a transition point [3]. “Coffee-bean” sign on plain X-ray is a feature of volvulus as seen in the case reported by Ghahremani *et al*. [5]. X-ray in our case was suggestive of IO, but the ultrasound was not informative as the colon was grossly distended.

Management of IO in pregnancy requires a multidisciplinary approach including obstetric and pediatric teams. Before laparotomy, resuscitation is vital with decompression, fluids, and correcting electrolytes [1]. Midline incision is ideal for best exposure, and a cesarean section can be done if the uterus is obstructing the operative field. Resection (sigmoidectomy) and colostomy are generally advised, but some surgeons perform primary anastomosis with or without colonic washout. However, this carries a risk of anastomotic leak due to the edematous and paretic bowel status posing future risks [2]. Hartmann’s colostomy was raised in our case because the colon was grossly distended and bowels were edematous due to the late presentation; on the contrary, Ghahremani *et al*. reported a sigmoidopexy because the bowel was not gangrenous and the pregnancy survived due to the early presentation [5].

Endoscopy can be another diagnostic and therapeutic option, allowing for derotation and decompression of the volvulus with success rates between 50% and 80% in nonpregnancy states. However, controversies of recurrences and failures of endoscopic management especially in the third trimester are still debatable [4].

## Conclusion

Sigmoid volvulus in pregnancy is a rare condition, and its early diagnosis is a challenge. A high index of suspicion and early surgical intervention are key for favorable maternal and fetal outcomes.

## Data Availability

All data used in this study are available from the corresponding author upon request.

## Abbreviations

NGT: Nasogastric tube
ICU: Intensive care unit
IO: Intestinal obstruction
